# How Shall We Blister?

**Published:** 1883-11

**Authors:** 


### How Shall We Blister?

I always use the cantharidal collodion. I shall paint a blister in this instance three by four inches, putting on three or four layers, and thenat once put over this a poultice. This is an almost painless way of raising a blister. I have never seen it produce strangury.—*Goodell in Phil. Med. Times.*

